# The Importance of the Uterine Ebb as a Factor in Pelvic Surgery

**Published:** 1877-04-20

**Authors:** 


### PAMPHLETS RECEIVED.

THE IMPORTANCE OF THE UTERINE EBB AS A FACTOR IN PELVIC SURGERY, by HORATIO R. STORER, M.D., of Boston, U. S.; former Vice President of the American Medical Association, and member of the Medico-Chirurgical and Obsterical Societies of Edinburg.

This is an able and inteaesting paper which we hope to notice more fully hereafter.

